# Supplementary material for: Meta-Analysis and Gene Set Enrichment Relative to ER Status Reveal Elevated Activity of MYC and E2F in the “Basal” Breast Cancer Subgroup
Source: PLoS One. 2009 Mar 9;4(3):e4710. doi: 10.1371/journal.pone.0004710 (PMC2650420; doi:10.1371/journal.pone.0004710)
Supplement: Dataset S1 — GSEA results from first screen. The file contains a zipped archive of the HTML results of the second GSEA screen. Reports for enriched or depleted datasets may be accessed from the “index.html” link within the my_analysis.GseaPreranked.1219975950625.rpt.zip ZIP archive. Enrichment plots are available for each of the 50 most enriched and depleted gene sets. (13.32 MB ZIP) [file pone.0004710.s001.zip › my_analysis.GseaPreranked.1219975950625.rpt.zip!//POMEROY_MD_TREATMENT_GOOD_VS_POOR_UP.html]

Details for gene set POMEROY\_MD\_TREATMENT\_GOOD\_VS\_POOR\_UP[GSEA]

|  || Dataset | zSco.ERneg.to.ERpos\_collapsed |
| Phenotype | NoPhenotypeAvailable |
| Upregulated in class | na\_neg |
| GeneSet | POMEROY\_MD\_TREATMENT\_GOOD\_VS\_POOR\_UP |
| Enrichment Score (ES) | -0.48314518 |
| Normalized Enrichment Score (NES) | -1.7375801 |
| Nominal p-value | 0.002967359 |
| FDR q-value | 0.052653693 |
| FWER p-Value | 0.924 |
Table: GSEA Results Summary

  

Fig 1: Enrichment plot: POMEROY\_MD\_TREATMENT\_GOOD\_VS\_POOR\_UP      
 Profile of the Running ES Score & Positions of GeneSet Members on the Rank Ordered List

  

| PROBE | GENE SYMBOL | GENE\_TITLE | RANK IN GENE LIST | RANK METRIC SCORE | RUNNING ES | CORE ENRICHMENT || 1 | PLOD1 |  |  | 334 | 4.848 | 0.0551 | No |
| 2 | NUP62 |  |  | 1684 | 2.770 | -0.0006 | No |
| 3 | MLL |  |  | 3274 | 1.805 | -0.0902 | No |
| 4 | C19ORF57 |  |  | 3584 | 1.671 | -0.0859 | No |
| 5 | REG1A |  |  | 3910 | 1.550 | -0.0847 | No |
| 6 | ETV4 |  |  | 4095 | 1.475 | -0.0741 | No |
| 7 | ACCN2 |  |  | 4973 | 1.165 | -0.1208 | No |
| 8 | DVL3 |  |  | 6559 | 0.631 | -0.2296 | No |
| 9 | CRMP1 |  |  | 8558 | -0.105 | -0.3782 | No |
| 10 | DYNLT1 |  |  | 8724 | -0.180 | -0.3876 | No |
| 11 | IRF3 |  |  | 8964 | -0.296 | -0.4007 | No |
| 12 | AIP |  |  | 9211 | -0.409 | -0.4124 | No |
| 13 | AP3B2 |  |  | 9803 | -0.725 | -0.4449 | No |
| 14 | LOC23117 /// DKFZP54 |  |  | 10091 | -0.904 | -0.4515 | Yes |
| 15 | ZNF167 |  |  | 10130 | -0.919 | -0.4392 | Yes |
| 16 | RRP22 |  |  | 10321 | -1.042 | -0.4362 | Yes |
| 17 | COL6A2 |  |  | 10549 | -1.205 | -0.4334 | Yes |
| 18 | SLCO2A1 |  |  | 10859 | -1.433 | -0.4329 | Yes |
| 19 | GPS2 |  |  | 11528 | -2.058 | -0.4491 | Yes |
| 20 | ECH1 |  |  | 11692 | -2.212 | -0.4247 | Yes |
| 21 | AMT |  |  | 11718 | -2.239 | -0.3896 | Yes |
| 22 | NDN |  |  | 12435 | -3.157 | -0.3912 | Yes |
| 23 | AES |  |  | 12534 | -3.313 | -0.3438 | Yes |
| 24 | APOD |  |  | 12582 | -3.398 | -0.2911 | Yes |
| 25 | USP4 |  |  | 12702 | -3.625 | -0.2400 | Yes |
| 26 | NBL1 |  |  | 12759 | -3.768 | -0.1819 | Yes |
| 27 | GTF3C1 |  |  | 12854 | -4.018 | -0.1225 | Yes |
| 28 | SRP54 |  |  | 13026 | -4.538 | -0.0602 | Yes |
| 29 | REEP5 |  |  | 13138 | -4.972 | 0.0137 | Yes |
Table: GSEA details [plain text format]

  

Fig 2: POMEROY\_MD\_TREATMENT\_GOOD\_VS\_POOR\_UP: Random ES distribution      
 Gene set null distribution of ES for **POMEROY\_MD\_TREATMENT\_GOOD\_VS\_POOR\_UP**
